# Utilising tracking technology to reduce the financial and patient care impact of lost ear, nose and throat equipment

**DOI:** 10.1308/rcsann.2025.0015

**Published:** 2025-04-08

**Authors:** J Bass, M Patel, K Kapoor

**Affiliations:** Surrey and Sussex Healthcare NHS Trust, UK

**Keywords:** Equipment, Lost, Costs, Tracking, Efficiency

## Abstract

**Introduction:**

The effective treatment of a patient in a timely manner requires specialist equipment, including in ear, nose and throat (ENT) services, where orifices require careful inspection. Otoscopy, flexible nasendoscopy, peritonsillar abscess drainage and nasal cautery are all common practices and cannot be successfully completed without the necessary equipment. These tasks all require expensive equipment that can easily be misplaced in a busy hospital. A paucity of equipment can delay patient assessment and negatively impact treatment, as well as reduce clinician efficiency and effectiveness. Here, we investigate the impact of equipment loss, and discuss a cost-effective solution to the problem.

**Methods:**

We surveyed ENT departments from 15 different trusts on how equipment loss impacted their patients, staff and their department financially. We also calculated the cost of equipment lost in our department over the course of a year. We subsequently placed trackers on our equipment and calculated the cost of lost equipment after 12 months.

**Results:**

Of the 15 trusts surveyed, 13 responded. Our survey demonstrated the average cost of lost items to be more than £4,900 per department, with concurrent delays in treatment and a reduction in patient-facing time. No equipment was lost after the trackers were placed.

**Conclusions:**

The use of commercially available tracking technology can help reduce the amount of time taken to locate equipment, prevent incurring higher costs and, most importantly, improve patient safety, with an estimated return on investment of more than 3000% and an increase in direct clinical care simultaneously.

## Introduction

Ear, nose and throat (ENT) teams use special equipment to fully and thoroughly examine patients, whether they are seen in a clinic, present through emergency departments or are inpatients. ENT clinicians carry out these examinations in a variety of settings across a hospital (wards, outpatient departments, emergency department, etc), which inevitably leads to equipment being mistakenly left at any of these locations. This can delay patient examination and treatment, affecting patient care.

With increasing demands on health services and the concurrent issue of finite funding for new equipment, prevention of waste and care of current equipment are paramount. Here, we discuss a survey of 13 trusts assessing how equipment loss has impacted their day-to-day activity, whether it has compromised patient safety and the financial implications for these departments.

We suggest a simple, cost-effective solution to this problem using readily available tracking technology, with the aim of reducing equipment loss, and therefore improving patient care.

## Methods

### Ethical review

As confirmed by our hospital research department, this study was exempt from formal ethical review. Data were collected and stored anonymously.

### Study population

We surveyed ENT departments from 15 different trusts on how equipment loss impacted their patients, staff and department financially. The 15 trusts were chosen to represent a variety of hospitals and practice types across England and were selected from a sampling frame of all 126 ENT departments in the country. The focus was on ensuring representation from different trusts ranging from district general hospitals to tertiary centres. These trusts were selected from geographical regions across England, from Manchester to Brighton.

The participants contacted were the on-call ENT clinician for that trust. They were contacted by telephone during normal working hours (8am to 5pm) and consented to receive a survey using a Google forms link during their on-call shift. Fifteen clinicians were contacted, of whom 13 participated in the survey. The two individuals who did not complete the survey despite reminders had initially agreed to participate. All participants were bleep holders on the on-call rota, and were regularly required to access to the equipment discussed.

We also calculated the cost of equipment lost in our department over the course of one year. We subsequently placed trackers on our equipment and calculated the cost of lost equipment after 12 months.

### Impact equipment loss has on patient care

Participants were asked how many times in the past year patient care had been significantly affected by equipment loss, as perceived by the clinicians. They were also asked how often they felt patient care was affected by equipment loss.

### Financial impact on the department

Participants were asked to name the most notable item their department had lost in the past year, and how many common, expensive pieces of ENT equipment had gone missing in the same timeframe. Recommended retail prices for replacing these items were obtained from the authors’ department. The prices used were those quoted by the suppliers to the department for replacing the items discussed in this study. Participants were also asked how much equipment loss impacts their departments financially.

### Time taken retrieving lost equipment

**Figure 1:**
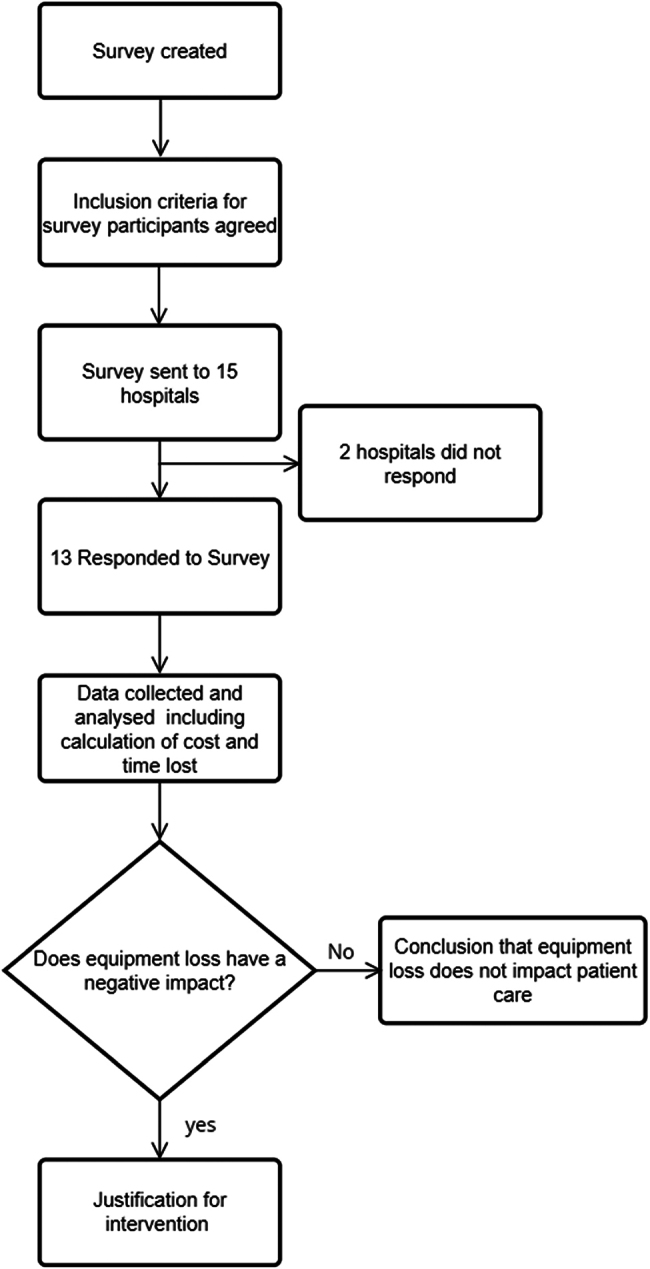
Flow chart demonstrating the methodology used for gathering survey responses

Participants were asked to estimate how much time is spent during the day retrieving lost equipment, and to estimate the amount of time that elapses until a lost piece of equipment is replaced (Figure 1).

## Results

Of the 15 participants invited to take the survey, 13 responded (86.67%). Of the 13 respondents, 3 were specialty registrar trainees and 10 were core surgical trainees or foundation level doctors.

### Impact of equipment loss on patient care

Over a one-year timeframe across 13 trusts, participants recalled 19 events in which patient care was significantly compromised (as perceived by the participants) because of a lack of equipment availability; one trust had five such incidents in a year. Only two respondents (15.38%) felt that none of their shifts were affected by missing equipment. Most trusts felt that there was a palpable impact on care, with 69.23% (9/13) believing that up to half of shifts were impacted to some degree.

### Financial impact of equipment loss on a department

Four respondents (4/13; 30.77%) estimated that expensive equipment went missing at least once a month, with eight departments (61.53%) estimating that equipment was lost at least once every 4 months. The average cost of these losses across the surveyed trusts was £4,904.77. The employee cost incurred for finding lost equipment per 24-h shift would therefore be between £9.88 and £23.32, based on an average Foundation Year 1 standard hourly pay rate of £18.10 in normal hours. At night and when locums are filling shifts, the time taken and cost per hour of the staffing are greater, and therefore the financial implications would also rise (Table 1).

**Table 1:** List of equipment lost

Item	No. of units lost	Cost per unit (including VAT) (£)	Total cost (£)
Headlight	18	1,896	36,024
Otoscope	10	330	3,300
ENT bag	7	87	609
Portable FNE light source	4	1,500	6,000
Bleep	3	300	900
Ambu^®^ endoscoscope screen	1	5,000	5,000
Light cable for FNE	1	561	561
Flexible nasendoscope	1	11,368	11,368
Total no. of items	45	Total (£)	63,762

ENT = ear, nose and throat; FNE = flexible nasendoscope

### Time spent without, retrieving or replacing equipment

Respondents were asked how long it takes to retrieve equipment when it does go missing. One respondent (7.69%) was able to retrieve the equipment on the same day; seven (53.85%) respondents were able to retrieve their equipment within 1 week, four (30.77%) within 2 weeks and one (7.69%) within 1 month.

**Figure 2:**
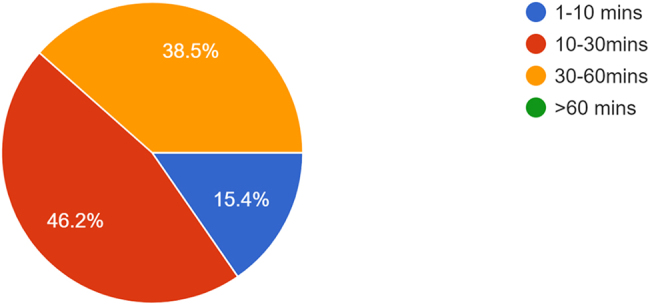
Amount of time respondents spent retrieving equipment over the course of a 12-h shift.

Respondents were questioned about the amount of time they spent retrieving equipment during a 12-h shift (Figure 2). On an average 12-h shift, two respondents (2/13; 15.38%) spend between 1 and 10min searching for equipment; six (46.15%) spend between 10 and 30min; and five (38.46%) respondents spend between 30 and 60min.

Respondents were asked: when lost equipment cannot be found, how long does it take for the item to be replaced? Two (15.38%) responded that if equipment is lost, it is replaced within 1 week; two (15.38%) responded that it is replaced within 2 weeks; four (30.77%) that it is replaced within 4 weeks; and five (38.46%) responded that it takes more than a month to replace expensive equipment. The median time expected for a piece of expensive to be replaced over the 13 trusts is 19.6 days.

### Clinician experience of equipment loss

When asked to recall specific instances in which patient care was affected, three respondents gave examples. One respondent described how their team would lose track of a piece of equipment because it was left on different wards, or in the emergency department.

Two respondents found that a lack of commonly available equipment was the main drain of time on their day, especially out of hours. If a piece of equipment was not readily available to them in the ENT bag, they would spend a lot of time trying to locate items from theatre, clinic or elsewhere.

### Respondents’ perceptions of financial impact on the departments

Twelve (12/15; 80.00%) of the surveyed clinicians responded to the statement: The cost of lost equipment impacts our department financially. Seven (7/12; 58.33%) respondents did not know how much equipment loss impacted their department. Four (4/12; 33.33%) respondents agreed that lost equipment did have an impact on their department, with only one respondent (1/12; 8.33%) feeling that missing equipment did not have an impact on their department financially.

### Suggestion

We undertook a quality improvement project using SMART methodology.

• S, Specific: our specific goal was to improve our equipment retention and availability.

• M, Measurable: we recorded equipment loss over a 6-month period after the introduction of our project.

• A, Attainable: our goal had a good chance of success, because the lead was in the team using this equipment and was supervised by an experienced consultant.

• R, Relevant: this project is relevant to ENT patient care, because equipment loss has an impact on patient outcomes.

• T, Timeframe: our survey demonstrated that equipment loss affected 61.54% of respondents within a 4-month period. A 6-month period was agreed to sufficiently demonstrate that this project would have a measurable improvement.

### Project proposal

**Figure 3:**
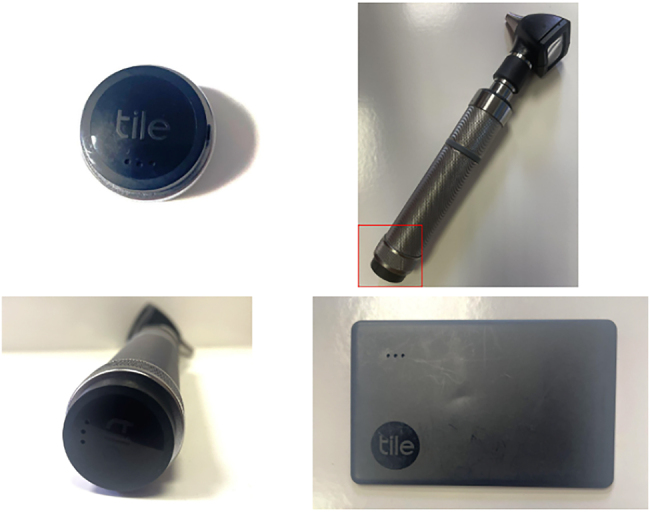
(a) Unattached Tile Sticker tracker. (b, c) Tile Sticker attached to an otoscope. (d) Tile Slim placed in the ear, nose and throat equipment bag.

Our team acquired Bluetooth tracking devices from the company Tile.^1^ These trackers work by connecting with the user’s phone via Bluetooth within the device’s range. If the device is not in range of the user’s phone, it can be tracked via another Tile user’s phone, as well as giving the user its last known location before becoming disconnected. The specific Tile products used in this paper were Stickers (Figure 3a–c), and the Slim Tracker (Figure 3d). Both devices have a 3-year battery life.

The Tile Sticker has a range of 250 feet, and the Tile Slim Tracker has a range of 350 feet. Two Tile Stickers cost £44.99, and the Tile Slim Tracker costs £29.99. There is no subscription charge, and the app required to track Tile devices is free. These devices were placed on our headlight, otoscope and ENT bag, over a 12-month period in our department. The outcome measured was how many of the tracked items were misplaced during this period.

Before the trackers were installed, our department lost two headlights, one otoscope, two ENT bags and one bleep, equating to a total cost of £4,542 in the preceding year. Trackers were installed on the replacements and tracked with an app by doctors on the on-call team.

Over a 12-month period, none of the tracked items were lost or needed replacing. Anecdotally, the ability to locate the missing item accurately reduced the time taken finding items; however, no formal evaluation of time saved was undertaken.

The total cost of the trackers was £74.98. Potential savings can be estimated from the cost of equipment lost in the previous year. This estimate is £2,271, giving a return on investment of 3,028.81%.

The availability of equipment enabled our team to examine patients in a timely manner, and thus ensured that they received better patient care than if this equipment was not available.

## Discussion

The patient is at the centre of everything a clinician does and we are always striving to improve patient outcomes and experience. Delays can negatively affect the quality of care a clinician can deliver, as well as the patient’s perception of the care they are receiving. Time spent locating and retrieving equipment reduces face-to-face clinical time with patients, decreases a sense of satisfaction for doctors and reduces the number of cases that can be managed over the course of a shift. This ultimately leads to a higher handover burden for subsequent team members.

Healthcare system resources are limited and poor utilisation can have a direct impact on patient care, both at an individual level and a population level. The lack of appropriate equipment can lead to delayed diagnosis and discharge, both of which have a direct impact on length of hospital stay, a key performance indicator when assessing care. This can lead to unnecessary inpatient admissions.

Another source of inefficiency in this context is the time spent searching for equipment; our study demonstrated that clinicians in these trusts spent 27.3min, on average, per department. If we extrapolate this across all 126 ENT units in the United Kingdom, 27.3min per shift per department means the National Health Service is losing 4.78 days of direct patient care every day across the nation. Financially, this is a cost of £1,037.67, which equates to £378,749.55 annually.

Inefficient use of resources also leads to poorer patient management, and may result in unnecessary admissions or prolonged inpatient stays in hospital. A simple example of this would be the treatment of a peritonsillar abscess (PTA), which can be effectively managed without overnight admission. This was demonstrated in a study undertaken by Al Yaghchi *et al*, whereby a protocolised treatment for PTA resulted in patients being safely discharged home the same day.^2^ These patients had a telephone follow-up and there was a high level of patient satisfaction, because they could be managed from home. The protocol used required a positive aspiration of the PTA.

To safely drain a PTA necessitates equipment not readily available throughout the hospital: a headlight, large bore needle or scalpel, local anaesthetic and a tongue depressor. Having to locate these items if they are missing has a palpable impact on the prompt initiation of treatment. The time taken to walk across a hospital to collect the requisite items is lost to patient-facing clinical time.

The loss of a simple headlight during a shift prevents a doctor from draining the PTA at the first consultation, leading to an overnight admission that impacts not only the patient, but also the available beds within a trust. The cost of a non-elective admission for a patient with a non-malignant ear, nose, mouth, throat or neck disorder (such as a PTA), ranges from £485 to £4,648, depending on severity and length of stay.^3^ By delaying aspiration on first consultation, we are incurring an added financial burden in addition to unnecessary admissions, because delayed management of a PTA can have serious consequences for the patient, requiring more severe, prolonged and invasive treatments to manage.^4^

Given that, among the trusts surveyed, the average time to replace expensive equipment was 19.6 days, this could impact many patients in the lead time taken to replace the necessary equipment to perform procedures such as PTA drainage.

Our survey reveals that most clinicians can spend a significant portion of their day retrieving equipment, instead of providing patient care. It is clear from the responses provided by doctors in training that there is a lack of appreciation of lost equipment and the direct impact on the department. Most clinicians in training are not privy to discussions about departmental budgets or the competing demands for equipment. Hence there may be a perception that equipment is automatically replenished when it is not present, as per their experience of peripheral venous cannulas, needles, ear speculums and wooden tongue depressors. This misconception can lead to carelessness, contributing to the loss of equipment, and in turn causing inefficiencies in patient care.

### Study strength and limitations

A strength of this study is that we assessed and demonstrated that the issue of equipment loss is not limited locally by surveying a range of trusts of varying sizes, serving different population demographics, and including both tertiary and secondary centres. The quantitative impact of equipment loss was easily measurable by calculating the financial cost of replacing lost equipment. This provided solid evidence of the benefit of our intervention by extrapolating the estimated losses from the previous year and calculating the return on investment.

Weaknesses of the study include a limited sample size, with only 13 respondents. This survey relied heavily on participant recall, and as such is very liable to recall bias. There was no more-formal assessment of the time saved after implementation of the trackers, nor was there a formal evaluation of the clinical impact of the intervention. This would be a good next step, although it would be difficult to create a control group for comparison. The number of clinicians of different grades was too small to draw any meaningful conclusions about how perceptions of the impact on patient care vary across these groups.

Participants were relied upon to recall equipment loss and the length of time teams were without pieces of equipment, which is also vulnerable to recall bias. A more reliable method would have been to have a log of equipment in each ENT department to accurately record the time a department was without specific items of equipment. To the authors’ best knowledge, use of equipment log books is not standard practice at present. It is the authors’ hope, however, that these trackers would reduce equipment loss and its impact on departments. Furthermore, the time that participants spent retrieving equipment was also based on retrospective recall. Future research could consider prospective data collection with use of smart watches or stopwatches for more accurate and robust recordings.

Finally, the quality improvement aspect of the study was performed in only a single centre, which may limit the ability to extrapolate the findings to other centres.

### In the literature

We found no studies in the literature specifically discussing the impact of lost equipment in the field of ENT; however, there are several studies and articles that discuss the impact of a lack of equipment and its effect on patient care.

The Scottish Health Technologies Group (SHTG) published an insightful study in 2022 exploring the potential of Bluetooth tagging for medical equipment.^5^ After performing a survey of different health boards, to ask about currently used medical equipment tracking, the SHTG performed a pilot study in an emergency department to explore how much time was being spent searching for equipment, and then compared the difference after the equipment was tagged using Bluetooth trackers. Their findings highlighted significant efficiency gains, notably an 80% reduction in the time staff spent searching for equipment. This improvement underscores the pressing issue of equipment loss as a major time and financial drain on healthcare systems.

Through a case-based analysis, the SHTG extrapolated these results, forecasting that a single emergency department could save an estimated £600,000 over a five-year period by implementing Bluetooth tagging.

Our study aligns with these conclusions, reinforcing the idea that equipment loss is a pervasive challenge in hospitals, with considerable financial and operational implications. Bluetooth tagging emerges as a promising solution to mitigate these inefficiencies, offering both time savings and substantial cost reductions.

A systematic review concluded that approximately 1 in 20 patients are subject to preventable harm during their medical care. Healthcare inefficiencies, such as equipment loss, delays in treatment and poor communication, are identified as significant risk factors contributing to this harm. These inefficiencies can lead to prolonged hospital stays, infections and other complications, exacerbating the negative impact on patient outcomes.^6^ A 2017 study discussing factors affecting working conditions in public hospitals mentions that poor infrastructure, including loss of equipment or poor maintenance, directly impacts patient care and service provision.^7^ This is particularly evident in public hospitals, where poorly resourced healthcare systems lead to insufficient medical supplies and inadequate facilities. These issues, in turn, contribute to poorer patient outcomes, reduced quality of care and increased challenges for healthcare workers, who struggle to deliver high-quality care. In addition, a lack of proper infrastructure and resources often results in delays in treatment, increased patient wait times and staff burnout. This further compounds the problem and creates a cycle of inefficiency that affects both patient satisfaction and staff morale.^8^ In the context of our study, the impact of equipment loss in ENT departments mirrors these findings. Our survey demonstrates how missing equipment not only compromises patient care, but also increases financial costs and wastes clinician time, leading to delays in diagnosis and treatment. Controlling both clinical and non-clinical influences to the best of our ability is key in reducing patient harm, as well as improving patient outcomes, and clinician experience.

### Impact of study

Most of us have experience in using tracking technology, be this to locate a misplaced phone or attaching such a device to our check-in baggage when going on a flight. Use of the same tracking technology can have a positive impact, not only reducing lost or misplaced equipment, but also simultaneously increasing direct clinical care. We have shown that over the period of a shift, participants recalled spending nearly 30min tracking down lost equipment. If tracking technology even halved the time to locate equipment, when mapped to the 126 ENT departments nationally, implementation would provide an extra 22,995h (958 days) of direct clinical care.

Given evidence of the impact of equipment loss, from financial costs to inefficient use of clinicians’ time and further burdens on an overstretched healthcare system, a simple solution to this problem is likely to benefit clinicians, organisations and, most importantly, patients in an uncomplicated and cost-effective manner.

While this study addresses lost time and cost, as well as impact on patient care, future work on this topic could compare these delays to a validated matrix for morbidity due to equipment loss.

## Conclusions

This study demonstrates that non-clinical factors, such as equipment loss, can significantly impact patient outcomes by delaying treatment and increasing inpatient lengths of stay. We present a simple, cost-effective solution to a problem that affects ENT teams across the region. Its implementation will enhance patient care and clinician experience while reducing workload burdens and allowing more time for clinical tasks. The incorporation of simple, over-the-counter technology in our department has had a dramatic effect on maintaining equipment levels, as well as reducing waste and costs in the ENT department. The adoption of such technology can yield a return on investment of more than 3,000%, in addition to enhancing effective direct clinical care for patients. A formal study exploring the time saved for clinicians locating and retrieving equipment would further strengthen the argument for its use.
